# The Rusty Eye: Ocular Siderosis Masquerading as Chronic Anterior Uveitis

**DOI:** 10.7759/cureus.101280

**Published:** 2026-01-11

**Authors:** Yee Jy Chai, Justin Yeak, Joan Marie Palikat

**Affiliations:** 1 Department of Ophthalmology, Hospital Sultanah Bahiyah, Alor Setar, MYS; 2 Department of Ophthalmology, Hospital Queen Elizabeth, Kota Kinabalu, MYS; 3 Department of Ophthalmology, Hospital Tengku Ampuan Rahimah, Klang, MYS

**Keywords:** chronic anterior uveitis, intraocular foreign body, ocular penetrating injury, ocular siderosis, pars plana vitrectomy

## Abstract

Ocular siderosis (OS) is a progressive, vision-threatening condition caused by a retained, iron-containing intraocular foreign body (IOFB). It can be easily mistaken for chronic anterior uveitis, especially in the absence of a clear history of prior ocular trauma. This diagnostic pitfall can delay appropriate management, worsening the visual prognosis.

A healthy 43-year-old man presented with recurrent redness, photosensitivity, and progressive blurred vision in the left eye, of one-year duration, worsening over the past three months. He was initially diagnosed with chronic anterior uveitis with elevated intraocular pressure, and commenced on topical steroids, cycloplegics, and antiglaucoma eyedrops. Examination revealed a mid-dilated, non-reactive pupil, with diffuse brown deposition over the corneal endothelium and anterior capsule of the lens. A subsequent follow-up visit revealed minimal improvement. Further questioning revealed a history of trauma two years prior, which was only self-treated with over-the-counter eyedrops. Siderosis bulbi was highly suspected in view of the mechanism of injury and the insidious onset of poor vision. Computed tomography of the orbit showed the presence of a well-defined hyperdense foreign body, producing streak artifacts near the vitreous base. The left eye recovered well after removal of the IOFB, with visual acuity improving to 6/15 three months postoperatively.

This case underscores the importance of considering OS in patients with atypical or refractory anterior uveitis, especially in the context of overlooked trauma. Early radiological evaluation for the localization of the retained IOFB and timely surgical intervention can lead to an excellent visual outcome.

## Introduction

Ocular siderosis (OS) is a condition resulting from a retained, iron-containing intraocular foreign body (IOFB) [1]. The ferrous IOFB undergoes dissociation, resulting in iron deposition with a higher affinity for intraocular epithelial structures. OS may occur from 18 days up to many years following ocular trauma [1]. Studies report the average patient age to be 22-25 years (range 18-67 years), with most OS patients being males involved in industrial accidents and a lack of eye protection at work leading to such injuries [2]. It is one of the uncommon causes for profound visual loss and can be potentially misdiagnosed as chronic anterior uveitis due to overlapping clinical signs [3]. This poses a diagnostic challenge, especially when a history of ocular trauma is not known or immediately recalled.

We report a case of a siderosis bulbi initially misdiagnosed as hypertensive, chronic anterior uveitis, illustrating the diagnostic challenges and highlighting the role of early imaging as well as the importance of detailed history taking in the identification of occult IOFBs.

## Case presentation

A 43-year-old otherwise healthy gentleman presented to the ophthalmology clinic with a one-year history of painless blurring of vision, intermittent redness, and photosensitivity in the left eye. His vision had deteriorated in the past three months, which prompted him to seek medical attention. During the first ophthalmology visit, his visual acuity was 6/9 in the right eye and hand movements on the left side.

**Figure 1:**
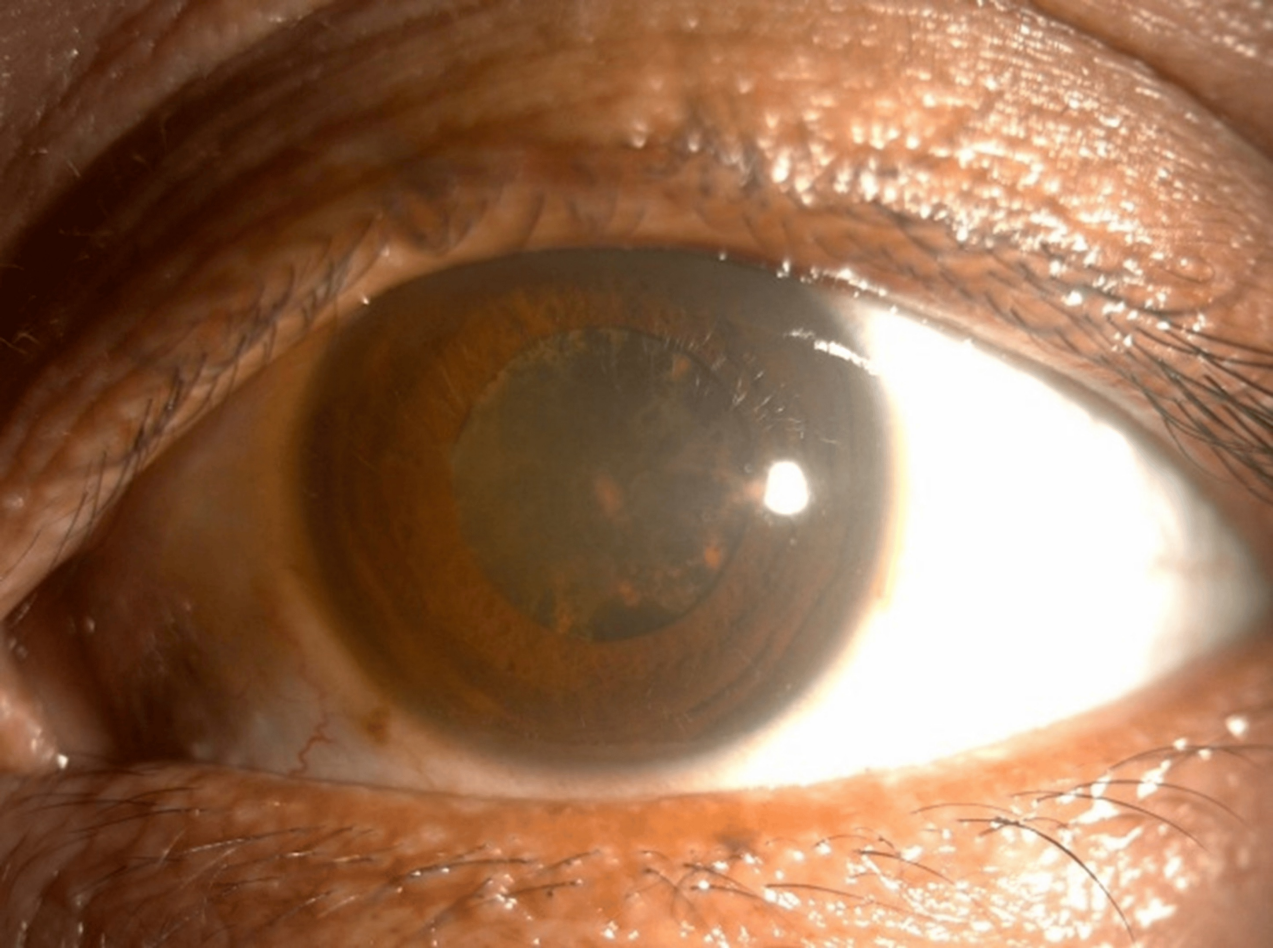
Anterior segment photo of the left eye revealing diffuse brownish deposition on the corneal endothelium and anterior capsule.

The left pupil was mid-dilated; however, no reverse afferent pupillary defect was detected. Light brightness and red saturation were equal in both eyes. The conjunctiva of the left eye was minimally injected, with the anterior chamber mildly flared, with diffuse brownish deposits distributed over the corneal endothelium and anterior capsule of the lens, rendering the left eye a poor fundus view (Figure 1). The intraocular pressure was raised at 26mmHg. The fellow eye examination was otherwise normal. He was diagnosed with chronic anterior uveitis with elevated intraocular pressure and commenced on topical steroids, cycloplegics, and anti-glaucoma eyedrops.

Unfortunately, his follow-up visit showed minimal improvement with persistent poor vision. Uveitis workup was otherwise unremarkable. Upon further history taking, he revealed that he had a workplace accident two years ago, where a small metal piece from a hammer hit his left eye while performing carpentry work. However, he did not seek medical treatment at that time as his eyes remained relatively asymptomatic after using over-the-counter eyedrops.

**Figure 2:**
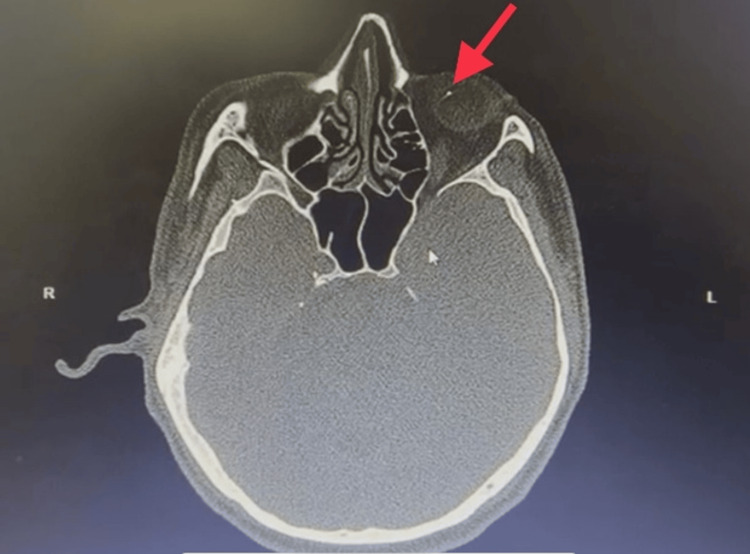
Axial view of the orbital computed tomography showing the presence of an intraocular foreign body (red arrow) located at the vitreous base.

In light of the history of the high-velocity metallic injury and the eye findings, siderosis bulbi was highly suspected. Computed tomography of the orbit showed the presence of a well-defined, hyperdense foreign body, which produced streak artifacts near the vitreous base (Figure 2).

**Figure 3:**
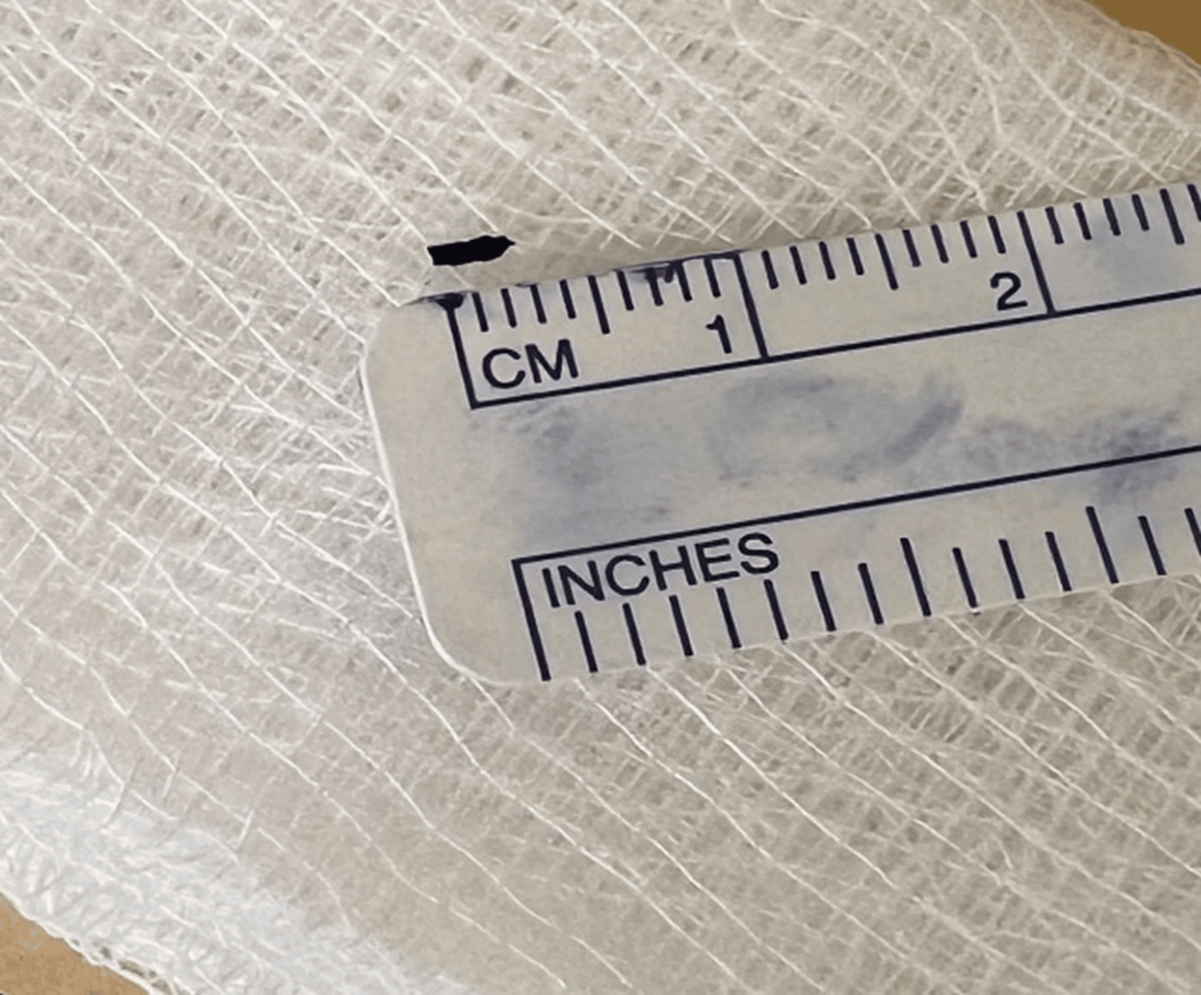
A metallic piece of debris measuring 2.5mm in length.

**Figure 4:**
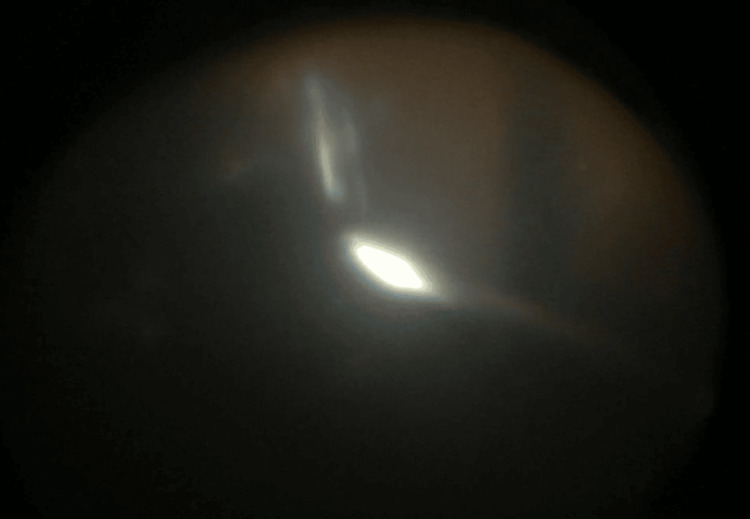
The intraocular foreign body was attracted and adhered to the intraocular magnetic probe.

**Figure 5:**
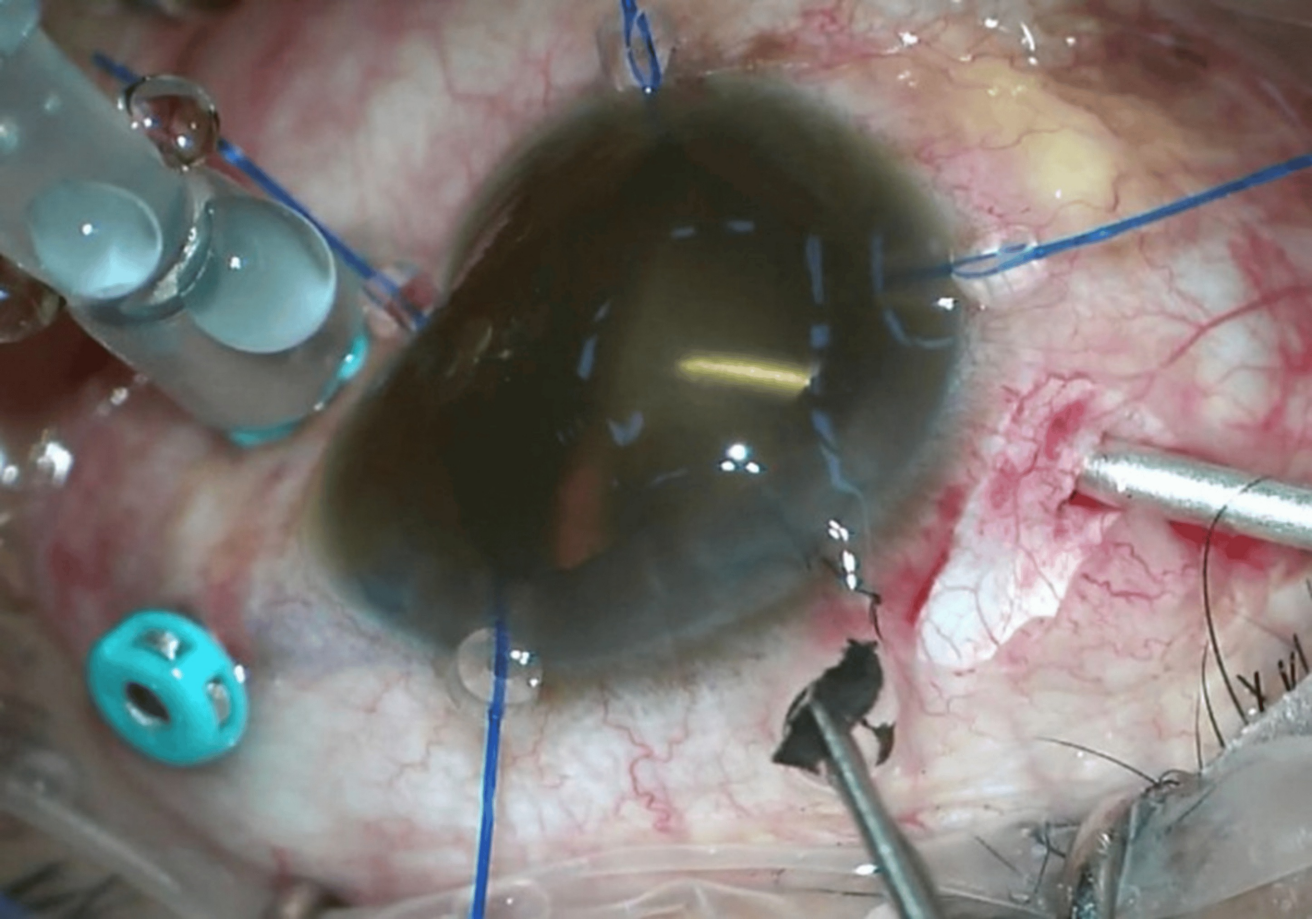
The intraocular foreign body was completely removed from the eye.

The patient was subsequently referred to the vitreoretinal team, where a combined cataract and vitreoretinal surgery was done to remove the IOFB. Pars plana vitrectomy was performed, and a metallic piece measuring 2.5mm in length (Figure 3) was removed with the aid of a magnetic probe (Figures 4, 5). A prophylactic barricade laser procedure was performed over the area of the retina where the metallic piece was embedded. Fortunately, a detailed internal search did not reveal other retinal tears or breaks, and the fundus examination did not show any pigmentary retinopathy changes.

**Figure 6:**
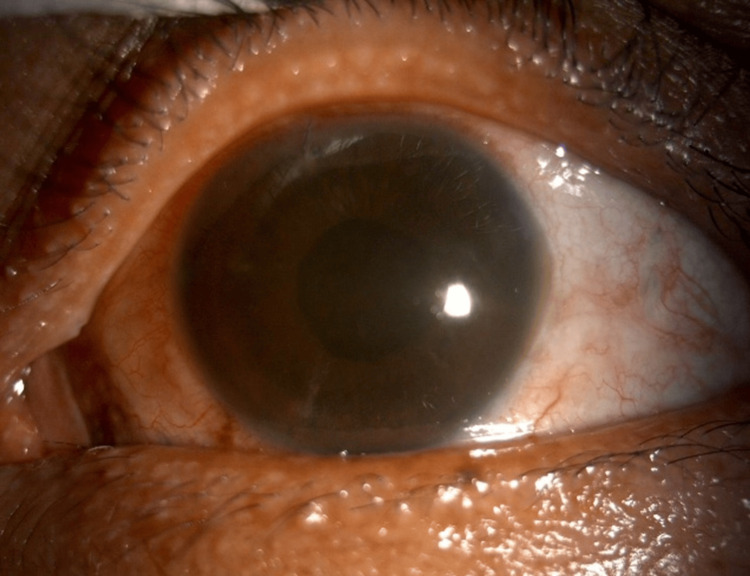
Anterior segment photo of the left eye three months after the removal of the intraocular foreign body, demonstrating improved corneal clarity.

During his follow-up visit, the patient was happy with the surgical outcome as he obtained a best corrected visual acuity of 6/15, with an intraocular lens implanted in the bag and a stable retina (Figure 6).

## Discussion

Ocular siderosis may develop as early as 18 days to years (even up to 12 years) after a penetrating ocular injury [1]. In fact, Pollack and Oliver reported a case of OS despite the removal of the IOFB [4]. Few studies have reported the average patient age of 22 to 40 years, with ages ranging from 19 to 67 years [5]. A vast majority of the patients are males (more than 90%), with most injuries attributed to occupational hazards [5,6].

Being an uncommon condition, the diagnosis of ocular siderosis requires a high index of suspicion as its clinical features may be subtle and may mimic chronic anterior uveitis. Its diagnosis is even more challenging if the patient fails to provide a positive history of ocular trauma, and clinical examination fails to reveal the presence of an IOFB. Our patient was initially diagnosed with chronic anterior uveitis due to the presence of ocular inflammation and failure in eliciting a positive history of ocular trauma. Similar misdiagnosis was also described by Mete et al. who reported a case of an undetected IOFB, with a two-year history, which also presented initially with marked anterior chamber inflammation and hypopyon formation [3].

Clinical presentations of OS can be widely variable depending on the location of iron deposition and time course. Heterochromia is the earliest sign of siderosis, more obvious in light-colored irises, which may be challenging to identify in those of Asian descent. In iron mydriasis, the pupil in the affected eye can be dilated and non-reactive, or with light/near dissociation [2]. Iron also tends to stain epithelial tissues in the corneal endothelium and anterior capsule of the lens, giving it a rusty or brownish hue as seen in our patient. Sometimes the lens is affected and becomes cataractous [7].

Secondary open-angle glaucoma can result from trabecular fibrosclerosis due to direct sedimentation of iron deposits onto the trabecular meshwork or secondary to albuminous aqueous production by the ciliary body [2,6]. Elevated intraocular pressure can also be attributed to siderotic uveitis, which was present in our case. As for the posterior segment, pseudo-retinitis pigmentosa can develop, showing features of arteriolar narrowing and sheathing with pigmentary retinopathy. Optic neuropathy with swelling of the optic disc and cystoid macular edema can also develop [8]. Fortunately, the posterior segment examination of our patient did not reveal any signs of optic neuropathy or retinopathy. These factors conferred a relatively good visual outcome after surgery. A complete ophthalmic evaluation is essential, especially a detailed anterior segment examination to look for any scarring or iris hole, which might suggest an entry site of the penetrating injury. The entrance wound, if possible, should be identified, and wound length should be determined to predict the IOFB trajectory and hence estimate the risk of retinal damage [2,5]. A detailed examination of the posterior segment should also be performed to look for any retinal tears and to localize the intraocular foreign body. In our patient, we could not identify any possible entry wound on the anterior segment examination, as it could have healed well due to the prolonged interval between the ocular injury and the patient’s presentation. This made the diagnosis of ocular siderosis even more challenging.

An IOFB can be detected via various imaging modalities [2,9]. Three-plane orbital CT without contrast is the gold standard for the identification of an IOFB, as it provides location and its size, with sensitivity up to 65% for an IOFB less than 0.06mm³ and 100% for more than 0.06mm³ size. In our patient, the IOFB was successfully located at the vitreous base via orbital CT. Ultrasonography modalities, such as B-scans and ultrasound bio-microscopy (UBM), provide two-dimensional images of the ocular and adjacent tissues with a high resolution, depending on the location of interest. IOFBs appear as a hyperechoic signal with comet-trail artefact [2]. If the IOFB is embedded in the retina, it can be captured by optical coherence tomography (OCT), which is useful for preoperative planning, to identify whether the IOFB is subretinal, intraretinal, or epiretinal [10].

Evaluation of retinal layer function can be done via an electroretinogram (ERG) [11] or an electrooculogram (EOG). The ERG is the gold standard for the detection of retinal toxicity caused by OS, typically exhibiting rod-cone functional abnormalities. In the context of our patient, unfortunately, evaluation of retinal function by an ERG and EOG was not done as these facilities were not available in our center.

The treatment plan for an IOFB depends on (1) chronicity of the IOFB, (2) location of the IOFB, and (3) whether there are clinical manifestations of OS. IOFB is indicated for removal if OS is present [2]. Otherwise, surgery can be delayed if the ERG does not show signs of OS and the IOFB is embedded in the subretinal layer or within a clear lens. In our case, the patient was fortunate to achieve a good visual outcome despite a delayed diagnosis, evident by the clearing of iron deposition in the ocular epithelial structures after vitreoretinal surgery. We postulate that the location of the IOFB might be one of the key prognostication factors to predict visual outcomes post-IOFB removal. First of all, the IOFB was located at the vitreous base, which is distant from the posterior pole; hence, the macula was relatively spared. Second, there were no retinal breaks or tears noted upon removal of the IOFB, and there were no signs of pigmentary retinopathy. This might indicate that the IOFB was probably entrapped in the vitreous without impacting the retina. Interestingly, Bloom et al. reported excellent visual outcomes one month after combined cataract extraction and pars plana vitrectomy in a case of OS due to retained IOFB [12]. Although an ERG three months post-surgery showed significant rod and cone abnormalities, a fundus examination did not show any sign of pigmentary retinopathy. This demonstrates the possibility of obtaining good visual potential post-IOFB removal if the retina appears normal on fundus examination.

## Conclusions

Ocular siderosis should be considered in all cases of unexplained unilateral chronic anterior uveitis, especially in patients with potential occupational hazards. Clinical features of siderosis bulbi upon presentation and a prior history of high-velocity eye trauma should raise high suspicion and prompt urgent radiological evaluation. The successful localization of the retained intraocular foreign body and timely surgical intervention can lead to an excellent visual outcome.
